# Plasma Leptin Is Increased in Intensive Care Patients with COVID-19—An Investigation Performed in the PronMed-Cohort

**DOI:** 10.3390/biomedicines10010004

**Published:** 2021-12-21

**Authors:** Anders Larsson, Miklós Lipcsey, Michael Hultström, Robert Frithiof, Mats Eriksson

**Affiliations:** 1 Department of Medical Sciences, Clinical Chemistry, Uppsala University, 751 85 Uppsala, Sweden; 2 Department of Surgical Sciences, Anaesthesiology and Intensive Care Medicine, Uppsala University, 751 85 Uppsala, Sweden; Miklos.Lipcsey@surgsci.uu.se (M.L.); michael.hultstrom@mcb.uu.se (M.H.); Robert.Frithiof@surgsci.uu.se (R.F.); mats.eriksson@surgsci.uu.se (M.E.); 3 Hedenstierna Laboratory, Department of Surgical Sciences, Uppsala University, 751 85 Uppsala, Sweden; 4 Department of Medical Cell Biology, Integrative Physiology, Uppsala University, 751 23 Uppsala, Sweden

**Keywords:** adipocyte, COVID-19, critical illness, cytokine, intensive care unit, leptin, SARS-CoV-2, SAPS3

## Abstract

COVID-19 has shaken the world and intensive care units (ICU) have been challenged by numerous patients suffering from a previously unknown disease. Leptin is a polypeptide pleiotropic hormone, mainly expressed by adipocytes. It acts as a proinflammatory cytokine and is associated with several conditions, known to increase the risk of severe COVID-19. Very little is known about leptin in severe viral disorders. Plasma leptin was analyzed in 222 out of 229 patients with severe COVID-19 on admission to an ICU at Uppsala University Hospital, a tertiary care hospital in Sweden, and compared to plasma leptin in 25 healthy blood donors. COVID-19 was confirmed by positive PCR. Leptin levels were significantly higher in patients with COVID-19 (18.3 ng × mL^−1^; IQR = 30.4), than in healthy controls (7.8 ng × mL^−1^; IQR = 6.4). Women had significantly higher leptin values (22.9 ng × mL^−1^; IQR = 29.8) than men (17.5 ng × mL^−1^; IQR = 29.9). Mortality at 30 days was 23% but was not associated with increased leptin levels. Neither median duration of COVID-19 before admission to ICU (10 days; IQR = 4) or median length of ICU stay (8 days; IQR = 11) correlated with the plasma leptin levels. Leptin levels in COVID-19 were higher in females than in males. Both treatment (e.g., use of corticosteroids) and prophylaxis (vaccines) have been improved since the start of the COVID-19 pandemic, which may contribute to some difficulties in deciphering relations between COVID-19 and leptin.

## 1. Introduction

Severe acute respiratory syndrome coronavirus 2 (SARS-CoV-2) causes the coronavirus disease 2019 (COVID-19), a pandemic responsible for more than a quarter of a billion confirmed cases, including over five million deaths as per WHO dashboard, dated 1 December 2021 [1]. Full blown COVID-19 may trigger a number of severe conditions, e.g., acute hypoxic respiratory failure, thrombo-embolic events, viral sepsis, bacterial superinfections, renal impairment, neurological disorders, and an overall extensive inflammatory reaction [2,3,4,5,6,7,8,9,10,11].

Leptin is a 16-kDa non-glycosylated hormonal protein, primarily synthesized by adipocytes [12]. Leptin is a pleiotropic hormone and a proinflammatory cytokine, influencing multiple endocrine functions, including both innate and adaptive immune responses [13]. Leptin may act as a link between obesity, metabolism, an increased inflammatory milieu, cytokine production, and a dysregulated innate immune response [14,15,16,17]. Leptin also promotes activation and chemotaxis of neutrophil granulocytes, regulates food intake through acting on the limbic system by stimulating dopamine uptake, and contributes to reactive oxygen species release [18,19,20].

Leptin is also associated with the formation of both arterial and venous thrombo-embolism [21,22], deterioration of kidney function, and increased morbidity and mortality in end-stage renal disease [23,24]. Factors that contribute to increased sodium reabsorption in obesity include adipokines, particularly leptin, which may contribute to renin–angiotensin–aldosterone system activation [25]. SARS-CoV-2 mediated leptin expression promotes inflammatory respiratory diseases, which link obesity and leptin as risk factors in COVID-19 [18,26,27,28]. However, there are several puzzling factors regarding leptin as a biomarker in COVID-19, e.g., both coronavirus SARS-CoV-2 and leptin exert inflammatory reactions involving the endothelial vascular surfaces, which could potentially act in synergy [27,29]. Furthermore, there is a mismatch in the quote between the anti-inflammatory adiponectin and the pro-inflammatory leptin in COVID-19 patients grouped depending on the severity of their disease [30].

The multitude of actions where leptin is involved is to a large extent similar to those occurring during infections with SARS-CoV-2. This raised our curiosity whether leptin could influence the clinical course in COVID-19 and, hence, be an independent predictor of outcome.

There are very few human studies on the interrelated effects of leptin signaling in infectious diseases, especially in severe viral infections, and there is a need for better understanding of circulating leptin [19]. Thus, we decided to evaluate whether leptin is increased in patients with COVID-19 and associated with basic clinical variables on mortality and morbidity.

## 2. Materials and Methods

### 2.1. Study Population

We investigated plasma levels of leptin in critically ill patients with COVID-19 included in the PronMed-study, approved by the National Ethical Review Agency (EPM; No. 2020–01623). In total, 229 patients diagnosed with COVID-19 were, during 14 March 2020–2 March 2021, admitted to the intensive care unit (ICU), a mixed surgical/medical ICU, at Uppsala University Hospital, a tertiary care hospital in Uppsala, Sweden. Twenty-five blood donors age: 47–68 years (median: 57 years; 19 males)) served as controls. According to the routines at our hospital, blood donors should be considered “healthy”, this means that disabling diseases or conditions reducing quality of life are not present. Certain conditions, e.g., malignancies (even cured), diabetes, or rheumatoid arthritis are not allowed. Certain well-treated disorders, (asthma, hypertension) without negative impact on exercise or daily activities are permitted. Before every blood donation is initiated, a health control is performed.

Informed consent was obtained from the patient, or next of kin if the patient was unable to give consent. The Declaration of Helsinki and its subsequent revisions were followed. The protocol of the study was registered a priori at (ClinicalTrials ID: NCT04316884). The STROBE guidelines were followed for reporting.

### 2.2. Blood Sampling and Analyses

Blood samples for analysis of leptin were obtained at admission to the ICU in 222 patients, collected in citrated tubes and centrifugated at room temperature at 1500× *g* for 10 min. The plasma samples were frozen at −70 °C until analyzed. Body mass index (BMI) was calculated as weight (kg) × height (m)^−2^.

### 2.3. Leptin Analysis

Plasma leptin levels (ng × mL^−1^) were analyzed using a commercial sandwich ELISA (DY398, R&D Systems, Minneapolis, MN, USA). Monoclonal antibodies specific for leptin were used as capture antibodies. Standards and undiluted samples were pipetted into the wells of microtiter plates and leptin was bound to the immobilized antibodies. After washing, a biotinylated antibody was added. After incubation and washing a streptavidin-HRP conjugate was added to the wells. After further incubation and washing steps a substrate solution was added. The development was subsequently stopped, and the absorbance was measured in a SpectraMax 250 (Molecular Devices, Sunnyvale, CA, USA) at 450 nm. The leptin concentrations in the samples were determined by comparing the optical density of the samples with the standard curve. The assays were calibrated against recombinant human leptin. The assay had a total coefficient of variation of approximately 6% and limit of detection was: 31 pg × mL^−1^.

### 2.4. Statistics

Data are presented as median and interquartile range (IQR). Differences between groups were investigated using Mann–Whitney U test with continuity correction. Correlation was calculated by the Pearson Correlation Coefficient Calculator. To describe the predictive value of plasma leptin on mortality, thromboembolism, artificial ventilation, renal replacement therapy, secondary infections, and critical illness neuropathy, areas under the curves were obtained from receiver operating characteristic curves (ROC). Statistica software, version 13.2 (StatSoft, Tulsa, OK, USA) was used for the calculations. Bonferroni adjustment was applied on morbidity and mortality. *p* < 0.05 was considered significant.

## 3. Results

### Study Cohort

**Figure 1:**
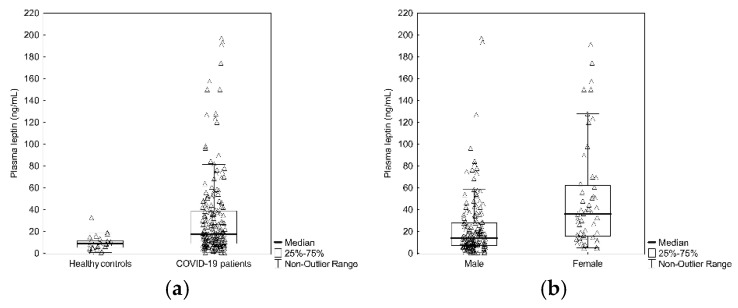
(**a**) Plasma leptin levels in ICU treated patients with COVID-19 vs. healthy controls (*p* < 0.001). (**b**) Difference in plasma leptin levels in ICU treated patients with COVID-19 based on gender (*p* < 0.001).

The patients were aged 24–86 years ((median: 64 years); IQR (Q3–Q1) = 19), 176 were males. Background data and pre-existing co-morbidities among the patients are seen in Table 1. Ten of the COVID-19 patients were current smokers and 61 patients were previous smokers. Median leptin level in plasma was significantly (*p* < 0.001) higher at ICU admission in COVID-19 patients (18.3 ng × mL^−1^; IQR (Q3–Q1) = 30.4), compared to healthy controls (7.8 ng × mL^−1^; IQR (Q3–Q1) = 6.4), (Figure 1a). Among patients with COVID-19, leptin was significantly higher (*p* < 0.001) in females (22.9 ng × mL^−1^; IQR (Q3–Q1) = 29.8) as compared to males (17.5 ng × mL^−1^; IQR (Q3–Q1) = 29.9) (Figure 1b). Median plasma leptin level in COVID-19 patients living 30 days after ICU admission was 20.3 ng × mL^−1^ (IQR = (Q3–Q1) = 32.1), whereas those who died within 30 days after ICU admission had median plasma leptin level at 16.7 ng × mL^−1^ (IQR = (Q3–Q1) = 23.6) (n.s.).

Median time with COVID-19 before admission to ICU was 10 days (IQR (Q3–Q1) = 4). Median Simplified Physiology Score (SAPS-3) on admission was: 53 (IQR = (Q3–Q1) = 12). There was no correlation between the duration of COVID-19 before admission to ICU and plasma levels of leptin (R^2^ = 0.0001), nor between leptin and length of stay at the ICU (median: 8 days); (IQR (Q3–Q1) = 11), (R^2^ = 0.009). Plasma leptin did not correlate with age (R^2^ = 0.0001), SAPS-3 (R^2^ = 0.0001), or BMI (median: 29); (IQR (Q3–Q1) = 8); (R^2^ = 0.0016).

The total mortality rate in COVID-19 at 30 days after ICU admission was 23% and ROC for leptin was 0.55. CIW was seen in 13% of the patients; ROC: 0.56. The incidence of thrombo-embolic events was 12.5% and ROC was: 0.54. Artificial ventilation was performed in slightly more than 50% of the patients; ROC: 0.51. Renal replacement therapy was performed in 12% of the patients (ROC: 0.51). Almost exactly 50% of the patients suffered from secondary infection (ROC: 0.56). Table 2 shows odds ratios, 95% upper/lower confidence limits, and *p*-values, respectively.

## 4. Discussion

Very little is known about plasma levels of leptin during severe viral infections demanding treatment in the ICU. Our main finding was that plasma leptin was higher in COVID-19 patients than in healthy controls, but without relation to the severity of COVID-19. This is partly in agreement with a previous study by Wang and co-workers [31], who focused on relations between cytokines, COVID-19, and BMI. They noted that leptin was higher in patients with COVID-19 and that there was a connection between the leptin levels and the progress of the disease. We did not find any relation between leptin and the severity of COVID-19 in the form of SAPS-3 and the plasma levels of leptin in our material. Additionally, there was no significant difference in plasma leptin levels between those who survived at least 30 days after ICU admission and those who did not. In fact, the median value of SAPS-3 among our patients was associated with a very high probability of mortality [32], suggesting that our patients were so sick that leptin had lost its possible predicting usefulness on mortality. Comparisons between these two studies are impeded, as there were 4–5% smokers in our material, whereas there were 60% current smokers among their COVID-19 patients [31]. Smoking cannot only upregulate the ACE-2 receptor utilized by SARS-CoV-2 [33], but smoking is also associated with increased plasma levels of leptin [34]. Furthermore, SARS-CoV-2 is known to upregulate the leptin signaling regulator SOCS-3 [35].

Leptin is also involved in sepsis. Plasma leptin levels were analyzed in a cohort of participants in a previous health survey and who later developed sepsis [34]. High leptin values predicted not only a future septic event but were also associated with a more serious one. After adjustment for BMI, this finding was significant in men only. At the time of onset of sepsis, increased leptin levels were associated with better survival in men, but not in women [36]. The gender-associated difference, which we observed in plasma leptin levels, has so far not been described in COVID-19 patients. Thus, the gender-associated effect of severe infections on plasma leptin does not seem to be limited to bacterial infections [36], but is also present in severe COVID-19, suggesting that immunologic, metabolic, and endocrine signaling of leptin in various severe infectious conditions are due to similar processes. Leptin levels may depend not only on gender, but also on time elapsed [37], as plasma leptin levels increased significantly in endotoxin-challenged humans at 24 h after, but not during a 7 h endotoxin infusion, administered at 2–3 ng × kg^−1^. The time aspect seems important, since leptin levels usually increase during the initial phase of sepsis, followed by a subsequent decline [38]. Genetic factors may also influence leptin levels, since mutation in the leptin-receptor gene is associated with obesity [39].

Adipocytes influence both the endocrine system and the immune response through several cytokine-like mediators known as adipokines, which include leptin. Using a cytokine array chip containing 174 proteins Wang and co-workers [31] found that overweight COVID-19 patients were more likely to have high levels of leptin. Elevated levels of circulating leptin may contribute to low-grade inflammation [40], making obese individuals more predisposed to increased risk of cardiovascular diseases, type II diabetes, or degenerative diseases [41,42].

There are some limitations to this study. This is a single-center study, where data was collected during a two-year period. During this time, the number of patients that fell ill with COVID-19 varied significantly. Additionally, knowledge about therapeutic management of COVID-19 patients was amplified. Furthermore, an increased number of vaccinated persons might have contributed to heterogenicity at the time of ICU admission.

We wanted to define a control group that reflects the population from a general perspective. Therefore, blood donors, permitting that a portion of their blood was used for scientific purpose, were chosen. It would have been desirable to have had these controls better characterized, but the ethical permit under which donor samples were acquired allows only for the registration of age and sex.

In addition, it should be taken into account that corticosteroid treatment had been initiated in slightly more than 10% of the patients at the time when blood samples for analysis of leptin were collected. Cortisol is a potent regulator of leptin expression in cultured human adipocytes [43]. We measured leptin in patients only at admission to ICU, but not in patients with COVID-19 who, for various reasons, were not ICU treated. Thus, we have no knowledge on any change in plasma leptin levels during the course of COVID-19 or different levels of leptin between these two different cohorts.

## 5. Conclusions

Leptin was higher at ICU admission in patients with COVID-19 as compared to blood donors, serving as controls. Plasma levels of leptin did not significantly differ between 30 day survivors and non-survivors in this cohort. Women had higher plasma levels of leptin than men, when admitted to ICU, which has never previously been described in COVID-19. Since very little is known about leptin in severe viral infections and the picture is hazy, future studies within this field may focus not only on progressive evaluation of adipokines during the course of COVID-19 but may also pay attention to subgroup analyses.

## Figures and Tables

**Table 1:** Background data and pre-existing co-morbidities (%) among the patients. Angiotensin converting enzyme inhibitor is denoted ACEi and angiotensin receptor blocker is denoted ARB.

Pre-Existing Condition	Frequency
Pulmonary disease	23%
Hypertension	57%
ACEi or ARB treatment	41%
Malignancy	10%
Heart failure	7%
Ischemic heart disease	13%
Thrombo-embolic event	10%
Steroid treatment	10%

**Table 2:** Plasma leptin in relation to clinical outcomes. CL = confidence limits. *p*-values were adjusted using the Bonferroni method.

Outcome	Odds Ratio	Lower CL 95%	Upper CL 95%	*p*	Adjusted Value
Critical illness weakness	1.01	1.00	1.01	0.03	0.18
Secondary infection	1.00	1.00	1.01	0.32	1.0
Renal replacement therapy	1.00	0.98	1.01	0.73	1.0
Mechanical ventilation	1.00	0.99	1.01	0.96	1.0
Thrombo- embolic event	1.00	0.99	1.01	0.80	1.0
Dead at 30 days	1.00	0.99	1.01	0.99	1.0

## Data Availability

The datasets used and/or analyzed during the current study are available from the corresponding author on request. This will in most cases also require an ethical permit.

## References

[1] World Health Organization (WHO). https://covid19.who.int/?gclid=CjwKCAjwuvmHBhAxEiwAWAYj-ImOFiU78cQgTvYgmWg3CPUHn9tYXyNgIck2p0uVpFfUCZL8YSz_1xoC6ksQAvD_BwE(20210726).

[2] Wiersinga W.J., Rhodes A., Cheng A.C., Peacock S.J., Prescott H.C. (2020). Pathophysiology, Transmission, Diagnosis, and Treatment of Coronavirus Disease 2019 (COVID-19): A Review. JAMA.

[3] Stattin K., Lipcsey M., Andersson H., Pontén E., Bülow Anderberg S., Gradin A., Larsson A., Lubenow N., von Seth M., Rubertsson S. (2020). Inadequate prophylactic effect of low-molecular weight heparin in critically ill COVID-19 patients. J. Crit. Care.

[4] Järhult J.D., Hultström M., Bergqvist A., Frithiof R., Lipcsey M. (2021). The impact of viremia on organ failure, biomarkers and mortality in a Swedish cohort of critically ill COVID-19 patients. Sci. Rep..

[5] Frithiof R., Bergqvist A., Järhult J.D., Lipcsey M., Hultström M. (2020). Presence of SARS-CoV-2 in urine is rare and not associated with acute kidney injury in critically ill COVID-19 patients. Crit. Care.

[6] Luther T., Bülow-Anderberg S., Larsson A., Rubertsson S., Lipcsey M., Frithiof R., Hultström M. (2021). COVID-19 patients in intensive care develop predominantly oliguric acute kidney injury. Acta Anaesthesiol. Scand..

[7] Frithiof R., Rostami E., Kumlien E., Virhammar J., Fällmar D., Hultström M., Lipcsey M., Ashton N., Blennow K., Zetterberg H. (2021). Critical illness polyneuropathy, myopathy and neuronal biomarkers in COVID-19 patients: A prospective study. Clin. Neurophysiol. Off. J. Int. Fed. Clin. Neurophysiol..

[8] Virhammar J., Nääs A., Fällmar D., Cunningham J.L., Klang A., Ashton N.J., Jackmann S., Westman G., Frithiof R., Blennow K. (2021). Biomarkers for central nervous system injury in cerebrospinal fluid are elevated in COVID-19 and associated with neurological symptoms and disease severity. Eur. J. Neurol..

[9] Gradin A., Andersson H., Luther T., Anderberg S.B., Rubertsson S., Lipcsey M., Åberg M., Larsson A., Frithiof R., Hultström M. (2021). Urinary cytokines correlate with acute kidney injury in critically ill COVID-19 patients. Cytokine.

[10] Huckriede J., Anderberg S.B., Morales A., de Vries F., Hultström M., Bergqvist A., Ortiz-Pérez J.T., Sels J.W., Wichapong K., Lipcsey M. (2021). Evolution of NETosis markers and DAMPs have prognostic value in critically ill COVID-19 patients. Sci. Rep..

[11] Bülow Anderberg S., Luther T., Berglund M., Larsson R., Rubertsson S., Lipcsey M., Larsson A., Frithiof R., Hultström M. (2021). Increased levels of plasma cytokines and correlations to organ failure and 30-day mortality in critically ill COVID-19 patients. Cytokine.

[12] Denver R.J., Bonett R.M., Boorse G.C. (2011). Evolution of leptin structure and function. Neuroendocrinology.

[13] La Cava A. (2017). Leptin in inflammation and autoimmunity. Cytokine.

[14] Wueest S., Laesser C.I., Böni-Schnetzler M., Item F., Lucchini F.C., Borsigova M., Müller W., Donath M.Y., Konrad D. (2018). IL-6-Type Cytokine Signaling in Adipocytes Induces Intestinal GLP-1 Secretion. Diabetes.

[15] Rebello C.J., Kirwan J.P., Greenway F.L. (2020). Obesity, the most common comorbidity in SARS-CoV-2: Is leptin the link?. Int. J. Obes..

[16] Di Renzo L., Gualtieri P., Pivari F., Soldati L., Attinà A., Leggeri C., Cinelli G., Tarsitano M.G., Caparello G., Carrano E. (2020). COVID-19: Is there a role for immunonutrition in obese patient?. J. Transl. Med..

[17] Fasshauer M., Blüher M. (2015). Adipokines in health and disease. Trends Pharmacol. Sci..

[18] Vallejos A., Olivares P., Varela D., Echeverria C., Cabello-Verrugio C., Pérez-Leighton C., Simon F. (2018). Preventive Leptin Administration Protects Against Sepsis Through Improving Hypotension, Tachycardia, Oxidative Stress Burst, Multiple Organ Dysfunction, and Increasing Survival. Front. Physiol..

[19] Birlutiu V., Boicean L.C. (2021). Serum leptin level as a diagnostic and prognostic marker in infectious diseases and sepsis: A comprehensive literature review. Medicine.

[20] Fernández-Sánchez A., Madrigal-Santillán E., Bautista M., Esquivel-Soto J., Morales-González A., Esquivel-Chirino C., Durante-Montiel I., Sánchez-Rivera G., Valadez-Vega C., Morales-González J.A. (2011). Inflammation, oxidative stress, and obesity. Int. J. Mol. Sci..

[21] Schäfer K., Konstantinides S. (2014). Mechanisms linking leptin to arterial and venous thrombosis: Potential pharmacological targets. Curr. Pharm. Des..

[22] Schäfer K., Konstantinides S. (2011). Adipokines and thrombosis. Clin. Exp. Pharmacol. Physiol..

[23] Briley L.P., Szczech L.A. (2006). Leptin and renal disease. Semin. Dial..

[24] Cohen G. (2020). Immune Dysfunction in Uremia 2020. Toxins.

[25] Hall J.E., do Carmo J.M., da Silva A.A., Wang Z., Hall M.E. (2019). Obesity, kidney dysfunction and hypertension: Mechanistic links. Nat. Rev. Nephrol..

[26] Vernooy J.H., Ubags N.D., Brusselle G.G., Tavernier J., Suratt B.T., Joos G.F., Wouters E.F., Bracke K.R. (2013). Leptin as regulator of pulmonary immune responses: Involvement in respiratory diseases. Pulm. Pharmacol. Ther..

[27] Lowenstein C.J., Solomon S.D. (2020). Severe COVID-19 Is a Microvascular Disease. Circulation.

[28] van der Voort P., Moser J., Zandstra D., Kobold A., Knoester M., Calkhoven C., Hamming I., van Meurs M. (2020). Leptin levels in SARS-CoV-2 infection related respiratory failure: A cross-sectional study and a pathophysiological framework on the role of fat tissue. Heliyon.

[29] Momin A.U., Melikian N., Shah A.M., Grieve D.J., Wheatcroft S.B., John L., El Gamel A., Desai J.B., Nelson T., Driver C. (2006). Leptin is an endothelial-independent vasodilator in humans with coronary artery disease: Evidence for tissue specificity of leptin resistance. Eur. Heart J..

[30] Di Filippo L., De Lorenzo R., Sciorati C., Capobianco A., Lorè N., Andrea Giustina A., Manfredi A., Rovere-Querini P., Conte C. (2021). Adiponectin to leptin ratio reflects inflammatory burden and survival in COVID-19. Diabetes Metab..

[31] Wang J., Xu Y., Zhang X., Wang S., Peng Z., Guo J., Jiang H., Liu J., Xie Y., Wang J. (2021). Leptin correlates with monocytes activation and severe condition in COVID-19 patients. J. Leukoc. Biol..

[32] Lemeshow S., Teres D., Avrunin J.S., Pastides H. (1987). A comparison of methods to predict mortality of intensive care unit patients. Crit. Care Med..

[33] Kashyap V.K., Dhasmana A., Massey A., Kotnala S., Zafar N., Jaggi M., Yallapu M.M., Chauhan S.C. (2020). Smoking and COVID-19: Adding Fuel to the Flame. Int. J. Mol. Sci..

[34] Eliasson B., Smith U. (1999). Leptin levels in smokers and long-term users of nicotine gum. Eur. J. Clin. Investig..

[35] Al Heialy S., Hachim M.Y., Senok A., Gaudet M., Abou Tayoun A., Hamoudi R., Alsheikh-Ali A., Hamid Q. (2020). Regulation of Angiotensin- Converting Enzyme 2 in Obesity: Implications for COVID-19. Front. Physiol..

[36] Jacobsson S., Larsson P., Johansson G., Norberg M., Wadell G., Hallmans G., Winsö O., Söderberg S. (2017). Leptin independently predicts development of sepsis and its outcome. J. Inflamm..

[37] Landman R.E., Puder J.J., Xiao E., Freda P.U., Ferin M., Wardlaw S.L. (2003). Endotoxin stimulates leptin in the human and nonhuman primate. J. Clin. Endocrinol. Metab..

[38] Tschöp J., Dattilo J.R., Prakash P.S., Kasten K.R., Tschöp M.H., Caldwell C.C. (2010). The leptin system: A potential target for sepsis induced immune suppression. Endocr. Metab. Immune Disord. Drug Targets.

[39] Farooqi I.S., Wangensteen T., Collins S., Kimber W., Matarese G., Keogh J.M., Lank E., Bottomley B., Lopez-Fernandez J., Ferraz-Amaro I. (2007). Clinical and molecular genetic spectrum of congenital deficiency of the leptin receptor. N. Engl. J. Med..

[40] Iikuni N., Lam Q.L., Lu L., Matarese G., La Cava A. (2008). Leptin and Inflammation. Curr. Immunol. Rev..

[41] Hou N., Luo J.D. (2011). Leptin and cardiovascular diseases. Clin. Exp. Pharmacol. Physiol..

[42] Allison M.B., Myers M.G. (2014). 20 years of leptin: Connecting leptin signaling to biological function. J. Endocrinol..

[43] Wabitsch M., Jensen P.B., Blum W.F., Christoffersen C.T., Englaro P., Heinze E., Rascher W., Teller W., Tornqvist H., Hauner H. (1996). Insulin and cortisol promote leptin production in cultured human fat cells. Diabetes.

